# Understanding fatherhood and its implications on the lives of male youth in Bangladesh: Findings from a nationwide mixed methods sexual and reproductive health study

**DOI:** 10.1371/journal.pgph.0003299

**Published:** 2025-03-31

**Authors:** Sameen Nasar, Abdul Jabbar, Syed Hassan Imtiaz, Subas Biswas, Mahmodul Hasan Shesheir, Sabina Faiz Rashid, Farzana Misha

**Affiliations:** 1 BRAC James P Grant School of Public Health, BRAC University, Dhaka, Bangladesh; 2 International Centre for Diarrhoeal Disease Research, Bangladesh, Dhaka, Bangladesh; Nagasaki University, JAPAN

## Abstract

Understanding of fatherhood amongst men greatly influences men’s roles and practices. There is limited information in the Bangladeshi context on the knowledge, perceptions, and practices regarding fatherhood of young males, which have a significant influence on their sexual and reproductive health (SRH). This study uses data from a nationwide mixed methods study on the SRH of young men in Bangladesh. Information on the study participant’s marital status, age at marriage, childbearing age, and financial and economic preparation was used. From 40 focused ethnographic case studies, we aimed to uncover fatherhood conceptualizations and practices. About 92% of the young married males reported their wives’ first pregnancies to be planned; approximately 13% had become a father before 18 years. There is an inverse relationship between reported household financial status and economic preparation for fatherhood. Ethnographies revealed that the understanding of family planning among male youth is limited to only birthing a child. Perceptions of fatherhood is contested between traditional norms of a strict disciplinarian and a globalized ideal, where, physical and emotional care to children in addition to financial resources is prioritized. Knowledge gaps still exist in realizing aspirations for fatherhood, which can be attributed to a lack of services for men’s sexual and reproductive health. As fatherhood impacts early childhood development, fertility decisions, and family management, it should be considered a priority within the health system of Bangladesh.

## Introduction

Parenting and parenthood initiation have persisted through human life and are shaped by sociological and historical contexts [1]. These contexts have been documented historically through classical literature [2], where systematic research by social scientists started relatively recently, focusing on mothers [1] and their roles in family planning and raising children’s lives across the world [3]. Although both motherhood and fatherhood are important for a child, research on fatherhood was limited in comparison. Fatherhood is the state of being a father, where, an influential book ‘Fatherhood: A sociological perspective’ provided the basis of an extensive description of fatherhood and the father; simply stating that fathers are critical to ensuring a stable family system [4]. Fatherhood as a research topic started after 1970, expanding to focus on ‘fathering’ (behaviour) and ‘fatherhood’ (cultural expectations) [3,5]. In recent years, research on the father, fathering, and fatherhood has extended worldwide [6], though the extent of research varies from country to country; for example, Bangladesh has a relatively narrow focus in fatherhood related research [7]. In addition, research on the perception of fatherhood in the indigenous communities can be found, though this is also limited [8].

Culturally, there are similarities and dissimilarities in the meaning and responsibilities of fatherhood. During the 21st century, the involvement of women in the workforce has prompted more men to be involved in childcare; for example, in the US, economic factors such as increased maternal employment, part-time and flexible work, and home-based work have led to increased parental responsibility, as mothers can earn relative to their counterparts [1]. Whereas in Asia, the nature of fatherhood and fathering are still quite diverse, that is, despite economic development across some of the countries, differences remain; for example, in Singapore, an advanced economy, greater sharing of parenthood was reported by fathers [1], contrasting with countries such as the Philippines and Vietnam, where there are delineations between roles of mothers and fathers, fathers having more of the provider role. On the contrary, fathers in Indian cultures tend to maintain distance from their children, and avoid being emotionally available to their children as they think sharing emotions will let them lose control over their children [9]. Chaudhary explained, Indian fathers are more involved in providing ethical guidelines, punishing rough behaviour, and controlling daily activities of their children, where in the western societies, fathers provide more attention in guiding their children to gain the necessary skills that help them to survive in the competitive job market. In Bangladesh, like India, strong family ties form the foundation of communities, with the elder males often playing a dominant role in family life, regardless of ethnic, cultural, or religious differences [10]. This contributes to the patriarchal norms that govern Bangladeshi society [11].

Here, fathers are expected to provide for the family and make all decisions pertaining to their children, such as selecting a school, hospital, or even a way of life [12]. Over time, the roles and responsibilities performed by the fathers have also changed in Bangladesh. Fathers belonging to an older generation perceived their primary responsibility to be meeting their children’s needs in terms of health and education [3]. In contrast, younger fathers believe that, the need to form emotional bonds is just as important as undertaking the same responsibilities performed by the fathers of the previous generations. In recent years, many urban fathers have reported that they are providing dedicated time for their children along with their wives/partners [13]. However, these urban fathers are unaware of the importance of such attachments for their children’s growth and development [12].

The ideas surrounding fatherhood have great influence on men’s roles and practices [14]. It also has broader influences on family relations and bonding. However, there is limited information in the Bangladeshi context on the knowledge, perceptions, and practices regarding fatherhood of young males, which have a significant influence on their risk taking, overall wellbeing, sexual and reproductive health behaviours and practices. The current generation of young Bangladeshi boys and men will become the fathers of the emerging generation, or already have stepped into fatherhood. Therefore, their understanding of fatherhood is important to provide structural and programmatic support for their SRH decision making, as well as their overall wellbeing. This paper uses data from a nationwide mixed methods study and analyses these concepts from the viewpoint of young men, uncovering their understanding of fatherhood, their practices, and detailing the gaps between perception and practice.

## Materials and methods

### Study design

This paper used data from larger nationwide research that aimed to understand the sexual and reproductive health and rights (SRHR) situation of young males (aged 15-24) in Bangladesh [15]. For this paper, we used the sections dedicated to collecting fatherhood related information in the main study, which followed a sequential cross-sectional mixed-methods design; a nationally representative quantitative survey followed by qualitative data collection using ethnographic methods. The study collected information from respondents regarding knowledge, perceptions, attitudes, behaviours, and experiences towards SRH; practices and experiences of SRH health-seeking behaviour including puberty, social norms, sex and sexuality, family and fatherhood, masculinity, and associated factors.

### Quantitative method: Cross sectional survey

We collected quantitative data from 370 clusters (289 unions for rural areas and 81 wards for urban areas; unions and wards are the lowest administrative units) of 370 sub-districts of 64 districts of 8 administrative divisions in Bangladesh. Using systematic random sampling, we conducted a survey amongst 11,012 young males aged between 15 to 24 years. This survey aimed to collect and examine knowledge, attitudes and practices, concerns, perceptions and experiences, health seeking behaviours, and service utilization and its associated factors regarding SRHR. All survey data were collected with the participant’s informed consent. We used a structured questionnaire for data collection through an electronic device (Samsung Tabs using SurveyCTO software; utilizing the Open Data Kit (ODK) platform―a tool widely used for collecting survey data). Face-to-face interviews were conducted by trained and experienced data collectors. Supervisors were assigned to monitor data quality, and provided feedback on a regular basis to ensure quality of data collection. For this paper, we used data related to socio-demographics, economic status of household, marriage, family planning methods, pregnancy and children related information, and factors associated with fatherhood as guided by previous literature.

The survey used a ‘Wealth Index’ to classify respondents’ households’ economic status. This was done using factor analysis. It was constructed using data collected on indicators that include household assets; livestock, agricultural land area estimation, electricity supply, household (exterior material, source of drinking water, roofing materials etc.), household appliances (electric fan, air conditioner, computer etc.), access to formal financial institutions (i.e., active bank accounts) etc. Initially, factor scores were generated for the total sample using a subset of indicators common to both urban and rural areas. Categorical variables were transformed into separate dichotomous indicators. Using a principal components analysis approach, a common factor score for each household was produced. Separate factor scores were calculated for urban and rural areas. To get the final factor scores for the entire sample, rural and urban scores were regressed on initial factor scores. Then each household was assigned a wealth score. The wealth score was divided into equal parts (quintiles) from lowest (poorest) to highest (richest); where, 1 = Poorest, 2 = Poorer, 3 = Middle, 4 = Richer, 5 = Richest.

### Qualitative methods: Focused ethnography

Following the survey, the qualitative study was conducted following a focused ethnographic approach. A focused ethnography deals with specific problems under a distinct context [16]. We conducted 40 case studies with young males aged from 15-24 years, who were purposively selected from four different geographical areas of Bangladesh:

a. Shyamnagar of Satkhira district (Rural coastal belt area)
b. Jagannathpur of Sunamganj district (Rural wetland (haor) basin area)
c. Rowangchhari of Bandarban district (Rural hilly area with ethnic communities)
d. Sholoshahar rail station slum of Chattogram City Corporation (Urban slum area)

The case study component in this focused ethnographic approach enabled us to get an in-depth understanding of the situation of male youth, their contexts, and factors that shape their SRHR perceptions, attitudes, behaviours, needs, as well as their health service seeking behaviours.

In addition, the research team also conducted eight focus group discussions (FGDs) with young males within the same age range, in-depth interviews (IDIs) with parents of young males, and key informant interviews (KIIs) with community influential persons and local health service providers. FGD participants were randomly selected from the respective communities, sometimes through acquaintances of case participants; these were conducted to understand the area and its culture, as well as the understanding of SRHR and its influence between peers. However, findings from FGDs were not used for analysis in this research and are not represented in the ‘Results’ section of this manuscript. All the qualitative interviews were audio recorded with the participant’s prior informed consent.

The case studies focused on individual life histories, which helped us to explore and understand individual perceptions, knowledge, expectations, choices, and practices regarding SRHR, also their fears, anxieties, confusions, and queries with SRHR. This method also supported us to uncover their needs, health seeking behaviours, sources of information, and challenges they faced when seeking formal or informal services. Most of these qualitative study participants were part of the quantitative survey, and later, were selected for the qualitative study based on their marital status, internet use, multiple sex partner history, drug use experience, and reported STI or sexual disease history, as well as their availability and willingness to participate in this study. In the focused ethnography, the research team collected information about respondent’s relationship with their father, their ideal characteristics of a father, planning regarding childbirth, preparation to become a father, and personality changes related to fatherhood.

### Analysis

Data were analysed using statistical software package STATA version(s) 15.0 & 16.0. Sampling weights were applied in all our analysis to provide a more accurate representation. We used univariate and bivariate analyses to explore fatherhood related data.

We collected information on the study participant’s marital status, age at the time of marriage, partner’s age at the time of marriage, children number, age at the birth of the first child, and whether they were prepared for being a father mentally and financially. We also observed the difference respondent preparation in urban and rural settings, and among different wealth status categories.

For the qualitative analysis, all the interviews were first transcribed and translated into English. We coded ethnographic data using ATLAS.ti (Version 8) software. Then the coded data were organized into data matrices on Microsoft Excel, this helped us to identify themes, types of information, and make comparisons between participants from different sites, therefore allowing us to conduct thematic analysis [17].

We aimed to uncover how the conceptualizations of fatherhood are constructed among young males, and how their practices reflect their conceptions. Additionally, we looked to understand the reasons behind their perceptions and practices.

### Ethical consideration

The study received ethical approval from the Institutional Review Board (IRB) of BRAC James P. Grant School of Public Health, BRAC University (2018-043-IR). Then, permission from respective authorities (government and local) was taken to gain access to the communities. Written informed consent was obtained from all the respondents. Participants were informed about their voluntary participation, privacy, confidentiality, anonymity, and the option of withdrawal from research. For respondents aged below 18-years; informed consent was also obtained from the parents or guardians following assent from the respondents. For respondents who were unable to read or write, verbal consent was obtained.

## Results

In this paper, the perceived notions of fatherhood among the young males will be explored, followed by their preferred characteristics for fatherhood, and how they are planning to achieve those characteristics. Lastly, practices as young fathers and, to what extent, their practices and perceptions converge will be explored. All qualitative results are exclusively from the 40 focused ethnographic case study interviews of respondents; other FGDs and interviews from community members were not considered for the qualitative analysis for this paper.

### Demographics

Among respondents for this study, only 8.78% (n=967) were ‘ever married’ (Table 1). The average age at the time of marriage of these respondents and their wives was 20 years and 17.7 years respectively (for both the quantitative & qualitative studies), with no significant difference between urban and rural respondents in the survey. We also found that 46.24% of ‘ever married’ participants had at least one child; their average age of becoming a father for the first time was 20.7 years. From 40 qualitative case study respondents, we found seven married participants; three of them were fathers of at least one child.

**Table 1:** Demographic information of survey & ethnography respondents.

Indicators	N (%)	
Marital Status	Quantitative	Qualitative
Unmarried	10,026 (91.05)	33 (82.50)
Married	967 (8.78)	7 (17.50)
Widow/widower	4 (0.04)	0.00
Divorced	14 (0.13)	0.00
Separated	1 (0.01)	0.00
At least one child (Ever married respondents)	456 (46.24)	3 (42.85)

### How young males understand family planning

A 20-year-old day labourer who is a father of a 2-year-old child reported:

*“I had a plan for a baby within the first year of our marriage. That was why we took our baby.”* He also provided an argument for early fatherhood, *“I heard that I cannot be a father after 2-3 years of marriage. That is why I became a father that soon.”*

Therefore, a question arises—how do young males perceive the notion of “planning” for fatherhood?

According to survey findings, about 91.8% of the young married males reported their wives’ first pregnancies to be planned. The average age for becoming a father for the first time was approximately 20.7 years. Also, around 13% of young married males had already become a father between the ages of 15-17 years. Young males from the poorest wealth quantile got married and became fathers earlier than the other groups. Moreover, the findings show higher educational attainment led to delaying marriage (21.4 years) (Table 2). Moreover, the young males who were economically active, on average, tended to marry a year earlier than the economically inactive respondents; almost twice as many had children (46.8%).

**Table 2:** Marital age and children related information of the married survey respondents.

	Age at first marriage (Average years)	Age of wife at first marriage (Average years)	Age when first became a father (Average years)	Children (%)
**Area**				
Rural	20.44	17.44	20.44	46.70
Urban	21.13	18.22	21.13	42.40
*p* **-value**				–
**Age Group**				
<18	15.50	13.60	15.00	13.00
18-20	18.70	17.00	18.14	22.00
21-24	20.30	17.80	20.92	50.80
**Wealth Index** ^a^				
Poorest	19.20	17.10	20.30	52.70
Poorer	20.10	16.90	20.80	44.40
Middle	19.60	18.80	20.60	51.40
Richer	20.70	17.20	20.90	36.00
Richest	21.00	18.90	21.20	34.40
**Educational Attainment**				
Less than primary	19.30	17.00	20.00	58.40
Primary Complete	19.30	16.40	20.60	63.40
Secondary Incomplete	19.70	17.00	20.90	37.80
Secondary Complete	21.10	18.10	21.11	24.10
Higher Secondary+	21.40	18.90	21.60	27.40
**Employment Status**				
Economically active	19.90	17.70	20.60	46.80
Economically inactive	20.60	16.70	21.10	26.90

^a^Wealth Index: 1 = *Poorest*, 2 = *Poorer*, 3 = *Middle*, 4 = *Richer*, 5 = *Richest*

Quantitative and qualitative results point towards the prevailing perception that family planning only involves the willingness to take a baby. If respondents felt confident enough to take a baby, they reported it to be ‘family planning’.

We also wanted to understand young males’ assurance with regards to fatherhood. Table 3 shows that, overall, 85.5% and 77.7% young males were mentally and financially prepared to be fathers respectively. There was approximately a nineteen-percentage point difference between rural and urban young males reporting mental and financial preparation for fatherhood (Table 3). Young males in households belonging to the ‘middle’ wealth bracket reported comparatively less preparation for fatherhood (Table 4). These questions were not asked to under–18 respondents; however, for the others, as age increased, the proportion of respondents reporting mental and financial preparation decreased.

**Table 3:** Preparation for fatherhood.

Indicators	Mentally prepared for fatherhood (%)	Financially prepared for fatherhood (%)
**Overall**	85.50	77.70
**Area**		
Rural	91.50	83.80
Urban	72.60	64.71
**Age group**		
<18	---	---
18-20	86.70	79.40
21-24	85.60	77.80
**Wealth Index** ^a^		
Poorest	86.00	71.20
Poorer	96.70	93.60
Middle	72.50	69.20
Richer	88.60	84.50
Richest	86.20	69.50
**Educational Attainment**		
Less than primary	91.00	80.20
Primary complete	69.90	57.80
Incomplete secondary	89.60	87.70
Secondary complete	92.20	87.50
More than secondary	91.00	89.00
**Employment Status**		
Economically active	85.30	77.30
Economically inactive	89.90	87.50

^a^ *Wealth Index (5 quintiles): 1 = Poorest, 2 = Poorer, 3 = Middle, 4 = Richer, 5 = Richest*

**Table 4:** Financial status associations & reported economic preparation for fatherhood.

	Economically Prepared for Fatherhood		χ^2^ (df)	*p*-value
	Yes % (n)	No % (n)		
**Principal Earning Member of HH**				
Self	61.70 (232)	63.75 (51)	4.9354 (3)	0.177
Father	31.38 (118)	28.75 (23)		
Mother	0.00 (0)	1.25 (1)		
Brother	6.91 (26)	6.25 (5)		
**HH Wealth Status (Wealth Index**^a^)				
Poorest	31.38 (118)	42.50 (34)	6.1368 (4)	0.189
Poorer	23.40 (88)	13.75 (11)		
Middle	23.14 (87)	18.75 (15)		
Richer	13.83 (52)	16.25 (13)		
Richest	8.24 (31)	8.75 (7)		
**Reported Monthly HH Income**				
Less than BDT 15,000	37.77 (142)	50.00 (40)	9.3992 (3)	0.024
BDT 15,001 – 25,000	43.09 (162)	43.75 (35)		
BDT 25,001 – 40,000	15.16 (57)	3.75 (3)		
BDT 40,001 – 70,000	3.99 (15)	2.50 (2)		

^a^ *Wealth Index (5 quintiles): 1 = Poorest, 2 = Poorer, 3 = Middle, 4 = Richer, 5 = Richest*

Further analysis between financial status measures and the reported perception of economic preparation for fatherhood shows that, about 62% of respondents who were the principal earners in the household were economically prepared for fatherhood (Table 4). However, when comparing against the household’s wealth status (as measured by the wealth index), majority of respondents from ‘poorest’ (31.4%) and ‘poorer’ (23.4%) households report being prepared for fatherhood, as opposed to respondents in the ‘richest’ households (8.2%); there was little to no evidence of relationships between these measures [χ2 (3, N = 376) = 4.93, *p* = 0.18; χ2 (3, N = 376) = 6.14, *p* = 0.19]. When compared to reported household monthly income, the data showed strong evidence of a relationship with reported economic preparation for fatherhood [χ2 (3, N = 376) = 9.40, *p* = 0.02]; respondents from households reporting total monthly income of BDT 25,000 (Bangladesh Taka) or less are more likely to report positively for economic preparation for fatherhood.

The following sections are solely based on the findings from the qualitative study. These include perceptions, attitudes and practices with regards to fatherhood.

### Perceptions of fatherhood

According to most participants, the “Father” resembled the most feared, honoured, and responsible person in the family, who is also the primary guardian of the family. He is the sole decision-maker in the family. Moreover, he is perceived to be an exemplary hard-working person, taking all necessary steps to ensure the safety of his family members, even if doing so puts his health at risk. A 20-year-old student from Bandarban said, *“I want to be like my father for any decision making, like taking risk and responsibility for the family.”*

### Father: The head and the guardian of the family―His words are final

Most participants echoed the perception that the father should be the ‘head of the family’. According to them, fathers play the most crucial role in deciding where the family is heading. He will make decisions on family planning, children’s education, and the marriage of his children. He will control everything in the family, and other family members will follow his directions. One 19-year-old married day labourer explained: *“If my father says ‘yes’ to something, we all will agree. After that, if my father says it will not happen, then it will not happen.”*

According to respondents, a father must maintain boundaries. This boundary will facilitate the power structure within the family. Out of 40 participants, over half (23) of them stated that, children should fear their fathers, which will result in them maintaining distance from their fathers. If they are not afraid of him, they may not be able to uphold social expectations of themselves, they claimed. In terms of the relationship between fear and respect, one 16-year-old day labourer said: *“Children have to be afraid of their fathers because a father can get respect if his children fear him.”*

However, there were differing views, few participants believed a father to be very close with his children, and understand them, their emotions, so children can share everything with their fathers. These respondents wanted the role of a father to be more liberal. Some of the respondents have already started shifting the perception of “father” from a purely hierarchical position to more open one. According to a 20-year-old student: *“If the parents have a good relationship with the child, then it is easy to know if the child has an imperfection or is good or bad, or has a problem. If a boy fears his father or is hesitant to say anything, then he will not know what his son wants from him.”*

From the accounts of our respondents, a dichotomy between the traditional father of the household and a more understanding father emerges; a fear based way of fatherhood that stems from being the sole decision maker as opposed to more of involved father. While fear-driven approach was reported necessary for discipline, a demand for a less hierarchical approach is borne out of experiences of respondents with father’s who were emotionally unavailable.

Regarding decision-making, some respondents also echoed a more open version of fatherhood. A 21-year-old respondent who works as a rickshaw puller said,

*“Everyone in the family should take the decision.”* Indicating an inclination towards joint household decision-making. Whereas another 17 years old student said*, “They will take their life decision according to their own will.”*

### Father—The provider: Can and will do anything for the well-being of the family

Most participants believe the father holds the prime responsibilities in the family. He must earn money and meet all household needs (food, accommodation, clothing, education etc.). One 18-year-old unmarried day labourer said, *“A father’s responsibilities are feeding his family members, giving clothes, and providing shelter to his family. A mother’s responsibilities include child care, cooking, and washing”*.

If a father cannot fulfil the needs of the family, he will be deemed a failure, both as a father and a man; people will call him ‘Kapurush’ (a coward). Therefore, a father must pursue any sort of work, no matter the risk. One 16-year-old unmarried rickshaw puller detailed: *“I saw many people work as painters on high rise buildings. They only use ropes and ladders. Why do they do it? Because of their families. A father must feed his family”*.

The quote provides an insight into why a father must cater to the needs of his children and the family he runs. For these respondents, the term ‘father’ resonates with responsibility. Therefore, an awareness of risks towards the whole family must be considered. As a result, decision making by a father needs to be calculated; this requires superiority in decision-making, pragmatism and consideration. According to one 20-year-old unmarried fishmonger:

*“A father must keep in mind that he has children and a wife. He should not take any risk that will make his children orphans”*.

While fatherhood clearly involves responsibility, for these respondents, it involves taking on personal risk. This is judged both societally and at the family level, as not being able to provide food, clothing and shelter is shamed upon. Furthermore, these notions generalise the separation of roles between a father and a mother within these communities, reinforcing rigid gender roles. This results in role based pressure on young men. This is because a father must cater to the needs of the family, and simultaneously take risks if needed, and ensure safety of family members from potential hazards associated with those risks. If a young man cannot fulfil all these expectations, he will deemed a failure within his community. Therefore, the idea of fatherhood for these respondents is constructed and maintained through the pursuit and achievement of these responsibilities.

### Attitudes towards fatherhood

According to the young male respondents, societal expectations of the position of fathers as providers and protectors of families and children. Consequently, all married participants expressed a strong desire to ensure their children received a formal education. They believed that proper education could enable their children to secure jobs, which would eventually support their own families and safeguard their futures.

Additionally, they felt that formal education could play a crucial role in preventing their children from engaging in or being involved in social crimes.

We also found that, almost all the participants wanted their children to be honest and avoid conflicts. According to them, someone with low inclination towards conflict is equivalent to a good human being. Moreover, they wanted their children to be known for being helpful human beings. Some participants even stated that their children should not reflect their personalities. A 16-year-old unmarried day labourer explained:

*“I want to be a real father. I want to educate my children. So that they can do something in life, he can be honest and good. He will not quarrel with anyone. He will not be like I am.”*

While respondents stated education as a necessity towards avoiding future social problems, like the 16-year-old respondent other respondents also reported that they did not want their children to be like them. This is a result of them recognizing the behaviours they have adapted from the parenting they received. This creates a distinction between respondents who viewed their fathers as role models, and those who did not want to emulate them.

### Fathers as role models

Close to half of the participants from the qualitative study reported wanting fear-driven respect from their children. They thought maintaining such a relationship would let them control their children, and prevent them from going towards a wrong path. This attitude was primarily reported in the Bengali communities. A 17-year-old unmarried student who feared his father explained:

*“I will behave like my father. If a child doesn’t fear his father, he can misbehave with his parents. That’s why it’s necessary to fear.”*

Another 19-year-old unmarried construction worker said, *“They should fear me. If they don’t fear me, they cannot become good human beings*.*”*

According to these respondents, fear was necessary for the development of children. This is predicated on the idea that, without fear there will be no discipline among the children in the family.

Regarding decision-making, some participants wanted to emulate their fathers. One 16-year-old unmarried rickshaw puller stated: *“When I have a family, I will have a child. I will take all the family decisions like my father does every day*.*”*

Another 21-year-old married day labourer in agreement stated:

*“The way my father manages our family, such as, how my father brought his kids up by tolerating many sufferings, I also want to apply these to raise my kids. Thus, the way I obeyed my father, my kids will also obey me the same way.”*

The quotes above reflect how these participants would prefer their family members to conform to their decisions. This establishes a prevailing idea amongst these respondents, which is that the amount of sacrifice with regards to jobs and earning should enable prime decision-making authorization within the household.

### Young males differentiating themselves from their fathers

Other participants wanted to maintain a friendly relationship with their children instead of fear-driven ties. They believed that their children should be able to share everything with them, allowing them to help them make important life decisions. They wanted to provide the support that they did not receive during their adolescence from their parents. These young males understood that they needed to be friendly guardians rather than administrators making orders. Many of them had experiences of unfriendly disciplinarian fathers. One 23-year-old unmarried private job–holder described:

*“My father is not so friendly. I was afraid of him. But I realized by myself that my son needed to share everything with me. Yes! After coming home, my son will tell me, ‘Father, I have seen a girl today, and I have liked that girl.’ My son will talk with me like a friend when he understands. I want my son to talk with me like this.”*

Some respondents would prefer to be fathers who are open to any type of conversation, as highlighted above. This would help them support their children and provide appropriate guidance. This emphasizes a division between traditional gender norms that influence ‘fathering’, with respondents realizing that what is required to be a ‘good father’ can be more than what they had experienced themselves.

These notions were developed by a reflection of experiences from respondents in age ranges of 15 to 19 and 20 to 24; relationships with respondents’ fathers often dictated their thoughts and aspirations with their own children. A respondent who became a father before the age of 18 reported when his father threw him out of the house.

*“My father threw me out of the house but I will not do this with my son. I will be free with my son as well monitor my son if needed.”*

This respondent expressed that he would not only be more open with his son, but would also is more vigilant. Another respondent reported a difficult relationship with his father, stating the relationship was driven by altercation.

*“I didn’t have a good relationship with my father. Say, we had an altercation, he would say, “You go, I will not go.” This kind of thing happened usually.”*

In addition to this, to this, he detailed the rigid nature of father-son relationships in Bangladesh, making comparisons with fatherhood in other countries.

*“Share everything with me. For example, in Bangladesh, a son cannot share anything with his father but I saw in other countries, especially in India, fathers and sons drink alcohol together....I will bring something for my son or daughter while returning home. When we enter our house, we say affectionate or adorable things to our children. A father should understand how his son runs his life. If I don’t, he will do something like boys taking drugs nowadays. Like that, he can become a drug addict.”*

The respondent also stated he would be attentive in his son’s life, helping him stay away from things such as drugs.

While traditional attitudes toward fatherhood portray fathers as decision-makers and breadwinners who command authority and instil fear in their children, this study found evidence of shifting perspectives among young males. Increasingly, they believe that fathers should take an active and emotionally engaged role in their children’s lives, moving beyond the conventional ‘breadwinner’ archetype. As a result, their approaches to fathering do not solely need to be based on their experiences, but a more reflective and constructive approach that goes beyond established gender norms in their communities.

This shift appears to be influenced by social networks and exposure to global ideas through education and digital media. These factors have encouraged a greater emphasis on the emotional aspects of parenting, with young fathers striving to build closer, more nurturing relationships with their children compared to previous generations.

### Preparation and practice for fatherhood

Most qualitative respondents reported that they did not have any formal knowledge of fatherhood. They thought they would learn through experience; the experiences of peers in their respective communities contributed to this perception. A 19-year-old respondent said:

*“I haven’t thought ‍about it (Fatherhood) at all. I didn’t even hear this from anyone before today. I just heard from you.”*

Most respondents had no plans to deal with childbirth. A 21-year-old taxi driver with a seven-month pregnant wife reported:

*“I have no plans at this moment. I don’t even feel anything now. I will plan it when my baby comes.”*

Also, majority of respondents did not have knowledge of antenatal care/perinatal care (ANC/PNC). The same respondent described:

*“I had no plan for a baby. My parents told me to take a baby, and I decided on taking a child. I told my wife. She agreed with me… I don’t know anything about it (ANC and PNC). I never went to the doctor with her. My mother always goes with her.”*

Regarding childcare, two out of the three fathers in the study reported not being able to provide sufficient time to towards their children due to work pressure. They did not think that child rearing was a father’s duty; they believed it to be the sole function of the mother. This is opposed to the respondents that subscribe to a more engaged role for a father. One young father stated:

*“How can I give time to my child? I must do a lot of outside work. I go out early in the morning and back at 10-11 pm.”*

The respondent detailed his working hours as a barrier to providing ideal amount of time towards his child.

However, another respondent father expressed a greater sense of responsibility, according to him:

*“I was careless to come home earlier or didn’t take much care. But after becoming a father, no matter how important work you have outside, you’ll always want to return home, as your child is at home and you want to see what he’s doing. That’s why I always try to come home early.”*

This father expressed that, despite the amount of work that he is required to do, it was equally important for him to engage with his child. This demonstrates changing attitudes towards a more emotionally engaged fathering role. Assuming fatherhood also gave these respondents a renewed sense of identity based on responsibility.

Amongst participants, there is also a prevailing notion that there is no need for any preparation for fatherhood—it is something to be attended to as it happens.

This was further shown while interviewing participants who assumed the role of fatherhood at the age of eighteen or younger. Based on the account provided by one of the participants, the decision to proceed with parenthood was influenced by the guidance provided by his parents.

*“I had no prior preparation for the impending birth of a child. I lacked financial resources. I adopted my child solely based on the instructions provided by my parents.”*

Despite no knowledge or money, the decision of the parents was prevalent and unquestioned, highlighting the importance of the household on decision making of our respondents.

However, one respondent who became a father after turning eighteen reported some level of financial preparation.

*“I was financially prepared before my child was born. I set aside around 4,000 to 5,000 BDT to ensure I could provide proper care and treatment for my child, even in case of unexpected situations.”*

In contrast, those who became fathers at the age of twenty-one or later generally reported being better prepared for starting a family, as they were more aware of the various associated costs. For instance, a twenty-one-year-old respondent described the challenges related to health services and doctor availability.

*“I was financially ready for it. I knew that the expectant mother requires specific attention since her conception. Money is the main problem. So, a decent amount of money like 20,000/-, 30,000/-, or 50,000/- should be kept at home. Suppose there’s no oxygen in our nearest hospital, or she might need to be taken to the city hospital at any moment. Also, competent doctors aren’t always available! That’s why money is very necessary. That’s why I have saved an additional 25,000 taka at home specifically for this purpose.”*

Older respondents with more work experience or educational attainment demonstrated a higher likelihood of understanding and adequately preparing for the challenges involved in raising a family. Despite these cases, the findings highlight the influence of family on decision-making, and the social and cultural importance placed on childbirth, which often leads to financial consequences for younger men in the household.

## Discussion

The evidence generated through this study shows that the conceptualization of fatherhood by the male youth of Bangladesh is still understood through traditional Bangladeshi family values; adopting responsibility, pragmatism and leading decision making within a household. While most respondents reported wanting to emulate a fear-based approach towards fatherhood, many reported the need for change; that is, a more friendly and attentive father. The study also finds that fatherhood for the male youth of Bangladesh is mostly based on experience, learned actions, and behaviour. This results in misunderstanding and knowledge gaps, which have implications on family planning and SRH; these include early marriage and childbirth. Quantitative results revealed that knowledge gaps still exist with relation to child bearing and economically supporting a family.

From quantitative findings we found that 91.8% of respondents reported their wives’ first pregnancies were planned. We also found the average age of becoming a father for the first time was only 20.66 years, and approximately 16% of married fathers were under the age of 18; according to Bangladeshi law, the minimum age for male’s marriage is 21 [18]. This not only reflects the non-adherence to marriage laws in communities, but also indicates risky sexual behaviours amongst adolescents in Bangladesh. Furthermore, the ability to bear a child is seen as a key evaluator for men in Bangladesh [19], contributing to the pressures of early childbearing. With regards to planning, there is an almost inverse relationship between financial status and reported economic preparation for fatherhood; those reporting higher household incomes, were less likely to report economic readiness for fatherhood.

Majority of qualitative respondents perceived a father to be strong, responsible, pragmatic, decisive, and an example for all household members, especially to his own children. They reported that fathers must arrange all the expenses for studies, medication, clothes, food, and take responsibility for all the decisions of the family. Whereas mothers are supposed to take care of the children and conduct household chores. This image of the father initiates a power hierarchy in the family [12], and in some cases, non-communication is seen between a father and a child. In this study, most participants reported that their fathers were responsible towards their families. They took risks to ensure a standard of living for their families. We find some respondents perceive their fathers to be roles models, while others did not want to emulate their fathers. Those that considered their fathers to be role models, hoped to support and become the sole decision-makers of their families. For the other set of respondents, the current image of the father is mostly fear-driven, which was reiterated through them reporting difficulties explaining their personal issues to their fathers. This contributed towards the changes in perceptions regarding fatherhood; young males have started to believe that a father can be friendly too. Some of the qualitative respondents described their ideal characteristics of a father―many of them were preparing themselves to be more open and supportive to their children, with the hope that their children would take the right decisions in life. However, under the planning theme, we found there is a lack of understanding on how to be a good father, and that family planning only equates to procreation, reflecting a gap in information at the household and community level. This may explain the lack of planning, which often does not match our respondents’ current or future aspirations to become a father. Furthermore, family pressures to get married and have children, as social reputation takes precedent for families, also compound the lack of planning. As a result, many respondents were left financially unprepared to start families, creating economic pressure.

Under the practice theme, we found that majority of respondents prioritise earning money rather than taking their wives for ANC/PNC services and childcare, as they think it is only the mother’s duty [20]. A qualitative longitudinal study carried out amongst teenage fathers in England, ‘Following Young Fathers’ (FYF), it was found that, most fathers conceived without planning, where only two fathers in the study had actively planned [21]. While it could be understood that that the lack of planning can lead to low levels of involvement in ANC/PNC by fathers, this study also identified the adoption of new paternal identities by young fathers; providing financially and materially has been seen as defining feature of young fatherhood. This was also seen in a book that details years of counselling experiences with young fathers in America, it finds that, most young fathers want to be emotionally and physically involved in relationships with their families [22].

For the context of Bangladesh, given the results of our study, we acknowledge the broader socio-ecological space, where the individual (adolescents and young men) are at the centre of personal and environmental factors [23], and are influenced by communities, institutions, and policies. The family unit is central to Asian life, as influenced by Confucian, Hindu, and Islamic teachings [24]. In Bangladesh, Islam is the dominant faith [25], and the Quran states that procreation is one of the most important functions of family life [24], further establishing childbearing as a key indicator of manhood in Bangladesh. However, traditional ideas of fatherhood are being contested, changes in perceptions of idealized fatherhood can be attributed to changing values that are intertwined with prevailing globalized ideologies; there are increased expectations to provide more physical and emotional care to children in addition to financial resources and moral teachings, as in many European and North American countries [24]. Despite these changes, interviews revealed that being a father is entirely based on experience, and as a result, our respondents had little to no planning, with no guidance from programmatic interventions or health facilities in making this transition. Therefore, what is learned and experienced in the family is typically emulated, resulting in early childbearing intentions and knowledge gaps. We found that, in addition to learned behaviour and knowledge within the family, relationships of respondents with their fathers dictated how they approached fatherhood, taking a more involved approach. This reveals a slow shift of gendered ideologies of parenthood in Bangladesh, as was seen in the FYF study [26–28]. However, our study found differences between respondents who became fathers in their teens as opposed to those who became fathers in their twenties; both groups wanted to differentiate themselves from their own fathers by taking on a more supportive role, financial and household planning varied greatly. Some teenage respondents were aware of the financial responsibilities; majority adhered to the decisions of their parents. In contrast, those who became fathers between after the age of twenty took more proactive steps towards family and financial planning. This is important to note as some of the early risk factors for early fatherhood include troubled father-son relationships, academic difficulties, school drop out, engagement in delinquent behaviour, gang activity or substance abuse, physical or sexual abuse, and psychiatric illnesses [29,30]. In addition to individual risky behaviour, it is equally important to acknowledge the wider socio-economic context in which individuals operate, as it has moderates intention at the household and individual levels.

Previous research in developed countries with respect to fertility choices revealed, unemployment, specifically unemployment of men, negatively affects fertility rates [31]. Whereas childbearing intentions across developing countries vary; studies conducted in Iran show that men from higher socio-economic backgrounds and higher current occupational status have less children [32]. A path analysis of selected men in Iran revealed that men’s age at marriage had the greatest effect on childbearing intentions, where, marriage at a younger age led to an earlier decision to have a child [33]. While indicators such as age, gender, GDP per capita [34] have shown effects on childbearing on different continents and countries.

It is important to consider that variables determining fatherhood or childbearing in one country may have different impacts based on the social context in which they occur. In the context of Bangladesh, younger age of marriage was seen to influence early childbearing, where, the higher the socio-economic background, the less likely a respondent is to report preparation for fatherhood. As fatherhood is understood as a social, moral and a relational process [35], which is mediated by religious and cultural traditions on the individuals―factors such as social reputation, myths and misconceptions, and family intentions greatly influence decisions on child bearing for male youth in Bangladesh, alongside economic factors, which have greater influence the greater the household income; evidenced by the inverse relationship between financial status and reported economic preparation for fatherhood. Furthermore, formal education levels of respondents and their households in our study did not impact decision-making. The lack of SRHR content in the national curriculum and the reluctance to teach SRHR [36], leading to lower knowledge and awareness, therefore reduced influence on decision-making.

The current notions and practices surrounding fatherhood amongst Bangladeshi male youth is at the intersection of globalized values and socio-economic conditions; where, the transfer of fathering knowledge across families is not equal across all districts. Prevailing motivations to live up to traditional norms still persist, evidenced by knowledge gaps in childbearing and family planning, despite some of the study respondents who reported an embracing of new values for fatherhood. While a study in rural South Africa found education and career settlement as a deterrent of early fatherhood [37], our data shows minimal differences between urban and rural Bangladesh in terms of marriage and childbearing, where only economic status of the household was seen to be a deterrent. Qualitative interviews shed light on the perception of family planning, which is the willingness to take a child. Respondents are not aware of other aspects of family planning, such as, child rearing and fathering—and as a result, there is larger space for misunderstanding and sub-optimal decision making. This puts adolescents and young men on a path towards misunderstanding SRH, therefore making them susceptible towards risky sexual behaviours, leading to spread of sexually transmitted infections [38]. Additionally, there are risks towards the loss of masculine self-esteem, which can lead to susceptibility towards suicidal ideation [25]; this is a cause for concern for young fathers who start families without adequate financial preparation.

### Future research and policy implications

There is a growing need for education about fatherhood, that is, the practical implementation of knowledge. While the focus of the field of Maternal and Child Health (MCH) and related fields have primarily been on mother’s health and behaviours and their impact on reproductive and infant health and development outcomes, there is growing evidence for the role of a father in the life of a child from birth [39,40]. Sexual and reproductive health services in Bangladesh are limited, primarily focusing on maternal health, where the importance of the father in SRH and family planning is mostly absent. Side-lining young fathers in such services has been shown to create a cycle of disengagement and distrust [27].

Findings from this study reveal information or fathering skills are not passed down equally within all communities, as most respondents were not aware of the concept. Additionally, there is no service provision assisting men on this transition. Therefore, given the findings in this study as it relates to misconceptions around family planning and fatherhood, as well as evidence on its importance on men’s health and mental health [40], it is critical that health services within Bangladesh acknowledge this underrepresented and underserved component of sexual and reproductive health and rights.

A limitation of our study was that the ‘Fatherhood’ section of this study was limited to married men in our sample, therefore, research on unmarried adolescents and young men and their perceptions and preparation for fatherhood is urgently required. Also, to further understand the patterns of fatherhood highlighted in this study, and the complex structure around fertility decision making in Bangladesh [38], more qualitative studies with ethnographic components need to be conducted.

Though there have been arguments for silence and sexuality in youth-parent relationships the Bangladeshi context [41], the increased use smart phones and access to the internet has changed the landscape of SRH [42], as was reported in the changing notions of fatherhood in this study. However, this also creates a greater scope for the misconceptions around SRH and fatherhood, also outlined in this study. Therefore, it is in the best interest of the male youth of Bangladesh, who are and will become fathers, to understand the importance and role of fatherhood with respect to health and SRH outcomes. There is a need to focus on the transition towards childbearing and fatherhood, which may be done through the national curriculum or through digital health services; focus on educating young boys on the details of fatherhood and family planning, as well as the application of compassion, which seems to be an emerging trend amongst majority of the youth in our study. Policymakers should acknowledge the understanding of fatherhood as an important and often overlooked area―which has implications on early births, child marriage, fertility rates, family planning and sexual health of men―and should prioritize it at health facilities under sexual and reproductive health services.

## Conclusions

Findings highlight that fatherhood is mostly understood through generational norms and knowledge, where there are some changing views within the current generation of Bangladeshi youth. However, gaps still exist in knowledge and practical issues related to fatherhood. As fatherhood impacts early childhood development, fertility decisions, and family management, it should be considered a priority within the health system of Bangladesh. Giving credence to this area has the potential to impact men’s sexual health, gender equity, and direction of future generations’ family planning activities.

## Supporting information

S1 Data Survey data. Ethnographic qualitative data.
(ZIP)
